# Supplementary material for: Factors that affect the utilisation of maternal healthcare in the Mchinji District of Malawi
Source: PLoS One. 2022 Dec 30;17(12):e0279613. doi: 10.1371/journal.pone.0279613 (PMC9803273; doi:10.1371/journal.pone.0279613)
Supplement: S1 File — (DOCX) [file pone.0279613.s001.docx]

Inclusivity in global research

PLOS’ policy on inclusivity in global research aims to improve transparency in the reporting of research performed outside of researchers’ own country or community and ensures that PLOS publications reporting global research adhere to high standards for research ethics and authorship. Authors of relevant research articles may be asked to complete the questionnaire below, which outlines ethical, cultural, and scientific considerations specific to inclusivity in global research. This questionnaire may be requested when researchers have travelled to a different country to conduct research, if research uses samples collected in another country, research with Indigenous populations or their lands, or if research is on cultural artefacts. Researchers travelling to another country solely to use laboratory equipment will not normally be required to complete the questionnaire. However, the questionnaire can be requested at the journal’s discretion for any submission – if you have been requested to complete this questionnaire by the PLOS journal you submitted to, please do so.

Please complete the questionnaire below and include this as a Supporting Information file with your manuscript. Note that if your paper is accepted for publication, this checklist will be published with your article in the supporting information files. Please ensure that you reference the checklist in the main body of your manuscript. We suggest adding a subsection ‘Inclusivity in global research’ to your Methods section and adding the following sentence: “Additional information regarding the ethical, cultural, and scientific considerations specific to inclusivity in global research is included in the Supporting Information (SX Checklist)”

The questions have been designed to be applicable to a wide range of study types, and there are subsections for both human subjects research and non-human subjects research. If any of the questions are not relevant to your research please mark them as “N/A” as appropriate.

**Ethical considerations, permits and authorship**

*This section is applicable to all research types.*

Provide details as to who granted permissions and/or consent for the study to take place in the Methods section of your manuscript. This should include the names of **all** ethics boards, governmental organizations, community leaders or other bodies that provided approval for the study. If individuals provided approval refer to these people by their role or title but do not list their name(s).

Reported on page number: 11

If there were any deviations from the study protocol after approval was obtained please provide details of these changes in the Methods section of your manuscript.
Did this study involve local collaborators that are residents of the country where the research was conducted or members of the community studied? If you do not have any authors from said communities, please provide an explanation for this below.

Reported on page number: N/A

JH worked with the MaiMwana Project in Malawi to establish this study and the LMUP team who collected the data used in this analysis was made up of local fieldworkers; one from each of the 25 geographical areas. No authors are from the local community as this is a seconday data analysis, however the MaiMwana Project, the local LMUP team fieldworkers who collected the data, as well as all the women who consented to take part in the study are thanked in the Acknowledgements.

Everyone listed as an author should meet PLOS’ criteria for authorship and all individuals who meet these criteria should be included in the author byline, rather than the acknowledgements. Authorship criteria is based on the International Committee of Medical Journal Editors (ICMJE) Uniform Requirements for Manuscripts Submitted to Biomedical Journals - for further information please see here: <https://journals.plos.org/plosone/s/authorship>.

**Human subjects research (e.g. health research, medical research, cross-cultural psychology)**

Did you obtain written informed consent from a representative of the local community or region before the research took place? How did you establish who speaks for the community? Details of written informed consent obtained from study participants should be reported separately in the Methods section of your manuscript.

Local approval to conduct the research in Mchinji District was given by the District Health Officer and the District Executive Committee. The research was linked to an existing surveillance system, which communities were already familiar with, and was introduced following a series of community sensitization meetings and discussions with village chiefs and local traditional leaders who gave their permission for the research to be conducted in their areas. Details of written informed consent are reported in the Methods section, within the ethics statement.

How did members of the local community provide input on the aims of the research investigation, its methodology, and its anticipated outcome(s)?

JH worked closely with the MaiMwana Project, a grassroots NGO from Mchinji District, Malawi to develop the research topic, methodology and questionnaires and to recruit local data collectors. MaiMwana had been working with the community for over a decade, were well-respected locally and in touch with the communities’ needs.

When engaging with the local community, how did you ensure that the informed consent documents and other materials could be understood by local stakeholders?

The information sheets and consent forms were developed in collaboration with MaiMwana who had extensive expertise of developing comprehensible conset processes. As part of the two-week training at the start of the project the data collectors were trained on the concept, importance and principles of informed consent through a combination of teaching, discussion and role-play. Information sheets could be read aloud and written informed consent, by thumbprint where necessary, was obtained from all participants before starting the interview.

Will the findings of the research be made available in an understandable format to stakeholders in the community where the study was conducted (e.g. via a presentation, summary report, copies of publications, etc.)? Please provide details of how this will be achieved.

Local dissemination of the primary findings of the research study took place in 2016. A series of feedback meetings were held, including with the data collectors, who were encouraged to share results with their local communities. A community event was held in each of the research zones, with village leaders, women and men invited and findings were shared verbally in the local language. A national dissemination event, with academics, relevant NGOs and government representatives, was held in Lilongwe in July 2016.

**Non-human subjects research using specimens/ animals collected as part of the study, or those housed in archival collections. Examples include archaeology, paleontology, botany and zoology.**

Did the permission you obtained from a local authority to perform the study include an agreement on access to outputs and benefit sharing? This may include procedures to enable fair distribution of the benefits and resources arising from the research performed. Please include any details of Prior Informed Consent and Benefit Sharing Agreements obtained. These may be required by field-specific regulations, for example the Convention on Biological Diversity (CBD) and the associated Nagoya Protocol.

N/A

If the material used in your study was imported, please A) provide the year it was imported and B) indicate whether permits were obtained to import/export the materials used, C) provide details of any permits obtained. If this information is not available, please indicate this.

N/A

If you used archival specimens, please state how the material used in your study was acquired by the institute it is held in and provide details of any permits obtained for the original excavations/ sample collection. If this information is not available, please indicate this.

N/A

How was the potential cultural significance of the materials collected in your study to local communities considered in your research design? Were Indigenous peoples and/or local researchers and institutions involved with archaeological excavations / collection of specimens? If so, please provide a description of their involvement.

N/A

If your manuscript includes photographs of human remains please indicate whether authors obtained permission from descendants or affiliated cultural communities to do so.

N/A
