# Supplementary material for: Factors that affect the utilisation of maternal healthcare in the Mchinji District of Malawi
Source: PLoS One. 2022 Dec 30;17(12):e0279613. doi: 10.1371/journal.pone.0279613 (PMC9803273; doi:10.1371/journal.pone.0279613)
Supplement: Attachment — Submitted filename: PLOS ONE Paper Review.docx [file pone.0279613.s002.docx]

**Reviewer Report**

**Title: Factors that affect the Utilisation of Maternal Healthcare in the Mchinji District of Malawi**

**Reviewer Report**

The study was supposed to examine the utilisation of maternal health and various factors affecting the utilisation of these health services.

The paper is written in a very crisp form explaining the background, objectives, methodology, and conclusion. The method chosen for this particular study is completely justifying in the context of the objectives given. Table 2 onwards provided in the research paper clearly depicts what they are meant for. The discussion part of the paper is also written in a well-mannered.

Only two suggestions to improve or revise the paper are as follows-

1. Table 1 can be more elaborated in terms of the sample size and results in one or two lines.
2. The authors have mentioned that the findings can be taken into consideration for policy making. If it is possible to mention one or two major findings which are strongly needed for the policy imperatives will enhance the value of this paper.

The paper can be considered for publication.

Thank you.
