# Supplementary material for: Factors that affect the utilisation of maternal healthcare in the Mchinji District of Malawi
Source: PLoS One. 2022 Dec 30;17(12):e0279613. doi: 10.1371/journal.pone.0279613 (PMC9803273; doi:10.1371/journal.pone.0279613)
Supplement: Attachment — Submitted filename: Response to reviewers.docx [file pone.0279613.s003.docx]

**Response to reviewers.**

| **Comments** | **Response to reviewer** |
| --- | --- |
| **Reviewer #1:** |  |
| The research work sounds a piece of scientific work, particularly for Malawi and similar setting. The table 1 presents good and clear picture of why some variables were chosen for further investigation. | Thank you. |
| Although the percentages were explained in the text, but it would be good to present in table with background characteristics and N against each background characteristics. | We have added a table listing the background characteristics, showing the n value for each characteristic. We have also done the same for the obstetric history information. |
| In discussion section, the findings state that “… living 5-10km away is associated with 0.6 times the odds of…”, the interpretation of the odds ratio needs to refine. | We agree that odds ratio can be hard to interpret, therefore, we have changed the wording of the findings in the discussion - it now discusses the findings in percentages, so is hopefully easier to understand (we have retained the odds ratios in brackets). |
| **Reviewer #2:** |  |
| The study was supposed to examine the utilisation of maternal health and various factors affecting the utilisation of these health services. The paper is written in a very crisp form explaining the background, objectives, methodology, and conclusion. The method chosen for this particular study is completely justifying in the context of the objectives given. Table 2 onwards provided in the research paper clearly depicts what they are meant for. The discussion part of the paper is also written in a well-mannered. Only two suggestions to improve or revise the paper are as follows-. | Thank you. |
| 1. Table 1 can be more elaborated in terms of the sample size and results in one or two lines. | We have updated Table 1, so it now includes the sample size and a brief summary of the results for each of the studies. We also removed one of the studies, replacing it with another similar study for which a precise sample size was available. |
| 2. The authors have mentioned that the findings can be taken into consideration for policy making. If it is possible to mention one or two major findings which are strongly needed for the policy imperatives will enhance the value of this paper. | We have added a section to the end of the discussion highlighting some suggestions for policy based off our results. As a result of this addition, we have slightly altered the order of points within the discussion to ensure the discussion flows well. Thank you for this suggestion – we agree that this change strengthens our paper. |

| **Academic editor’s Comments** | **Response** |
| --- | --- |
| Please ensure that your manuscript meets PLOS ONE's style requirements, including those for file naming. | We have gone through and checked that our manuscript meets PLOS ONE’s style requirements and have named the files as directed.   - Edited the subheadings to be sentence case. - Added supporting information section to end of manuscript - Added DOIs to references |
| Please include a complete copy of PLOS’ questionnaire on inclusivity in global research in your revised manuscript. | A completed copy of PLOS ONE’s questionnaire on inclusivity in global research is included as a supporting information file - referenced in the main manuscript under a subsection ‘Inclusivity in global research’ within the Methods section. |
| Your ethics statement should only appear in the Methods section of your manuscript | We have added an ethics statement to the start of the Methods section. |
| Please review your reference list to ensure that it is complete and correct. | We have reviewed our reference list and can confirm that it is now complete and correct. We have made some changes to our references list   - Replaced Ovikuomagbe, 2017 with Ononokpono et al., 2014 as detailed information regarding sample size was available. - Removed four duplicate reference (Rai et al., 2013, Chakraborty et al., 2003, Singh et al., 2014, Tsawe et al., 2015). - Removed Mubangizi, 2016 as poster presentation not publication (didn’t replace as already had other references saying similar things). - Reordered the reference list to reflect the change in the order of discussion. - Added DOIs to the references |
| While revising your submission, please upload your figure files to the Preflight Analysis and Conversion Engine (PACE) digital diagnostic tool. | We uploaded our figures to PACE and can confirm that they now meet the PLOS ONE’s requirements. |
